# The epidermal growth factor receptor (EGRF) in lung cancer

**DOI:** 10.1186/s40247-015-0013-z

**Published:** 2015-02-24

**Authors:** Enric Carcereny, Teresa Morán, Laia Capdevila, Sara Cros, Laia Vilà, Maria de los Llanos Gil, Jordi Remón, Rafael Rosell

**Affiliations:** Catalan Institute of Oncology, Hospital Germans Trias i Pujol, Universitat Autónoma de Barcelona, Department of Medicine, Badalona, Barcelona Spain; Hospital Santa Tecla, Tarragona, Spain; Hospital de Granollers, Barcelona, Spain; Hospital de Mataró, Barcelona, Spain; Cancer Biology & Precision Medicine Program, Catalan Institute of Oncology, Germans Trias i Pujol Health Sciences Institute and Hospital, Campus Can Ruti, Badalona, Barcelona Spain; Fundación Molecular Oncology Research, Sabino Arana 5-19, Barcelona, Spain

**Keywords:** Lung cancer, Epidermal growth factor receptor, EGFR, Tyrosine kinase inhibitors, TKI

## Abstract

In the last decade, important advances have been made in understanding of cancer biology, particularly non-small-cell lung cancer (NSCLC) with the discovery of oncogenic drivers of the disease. The epidermal growth factor receptor (EGFR) gene and its pathways was the first oncogenic driver discovered to be mutated and treatable in lung cancer. Treatment with EGFR tyrosine kinase inhibitors (TKIs) is the standard of care for molecularly selected *EGFR*-mutant patients, while its role in unselected lung cancer patients is nowadays controversial. This review will provide an overview of the EGFR pathway and options for its treatment of lung cancer.

## Introduction

The majority of non-small-cell lung cancer (NSCLC) patients are diagnosed with locally advanced or metastatic disease, meaning chemotherapy is the treatment of choice. However, despite advances, treatment with chemotherapy offers an overall survival (OS) benefit usually restricted to only a few months [1-3]. However, the outlook in NSCLC has changed significantly in the few last years. Lung cancer comprises a group of diseases with distinct molecular profiles and sensitivity to different treatments. It is mandatory therefore to determine and classify NSCLC molecular subtypes and to develop molecular diagnostics to identify them.

Our understanding of the role of molecular alterations in NSCLC has increased in recent years, leading to the discovery of driver alterations and the development of targeted therapies which has increased survival for a small, but significant group of patients. Mutations in the epidermal growth factor receptor (EGFR) gene and rearrangements of the anaplastic lymphoma kinase (*ALK*) gene are markers for determining the appropriate treatment for advanced NSCLC [4] and have now passed into routine clinical use as predictive biomarkers. Other potential predictive biomarkers such as ROS1, BRAF, HER2 or MET have also been identified and efforts are underway to target them with novel drugs.

## Review

### EGFR biology in lung cancer

The EGFR gene is located on chromosome 7p12–13 and belongs to a family of cell membrane receptor tyrosine kinases that include EGFR (ERBB1), HER2/c-neu (ERBB2), HER3 (ERBB3) and HER4 (ERBB4). Among these HER family members, EGFR and HER2 are the most commonly altered receptors in cancer. These receptors are single amino acid chain proteins that possess an extracellular ligand binding domain, a single hydrophobic transmembrane domain that is involved in interaction between receptors, and an intracellular domain with tyrosine kinase activity [5].

**Figure 1:**
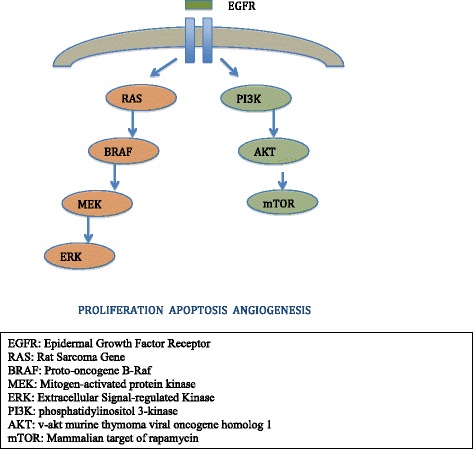
**Epidermal growth factor receptor pathway.**

EGFR activation can be induced through autocrine or paracrine ligands with different affinities for the ErbB receptors [6]. There are six major EGFR ligands including: epidermal growth factor, transforming growth factor alpha, heparin binding EGF, betacellulin, amphiregulin and heregulin. In EGFR, ligand binding induces a conformational change that facilitates receptor homo or heterodimer complexes among four receptors, thereby resulting in activation of EGFR tyrosine kinase activity. This autophosphorylation leads to downstream activation and signaling by several other proteins through their own phosphotyrosine-binding SH2 domains, initiating several signaling transduction cascades important for tumor cells, principally the RAS/RAF/MAPK pathway which plays an important role in regulating cell proliferation, migration and differentiation, and the PI3K/AKT pathway which controls cellular survival and antiapoptotic signals [7] (Figure 1). EGFR signaling pathways play an important role in the development of malignancy through modulation of cell cycle progression, inhibition of apoptosis, induction of angiogenesis and promotion of tumor cell motility and metastasis [8]. EGFR is known to be expressed more abundantly in malignant than in normal tissue, including 40–80% of NSCLC.

Several mechanisms lead to aberrant receptor activation, including: receptor overexpression, gene amplification, activating mutations, overexpression of receptor ligands, and/or loss of their negative regulatory mechanisms.

### Targeting EGFR in unselected populations

Interest in developing anti-EGFR treatments for specific tumors such as colon cancer and NSCLC has led to the development of two classes of drugs: monoclonal antibodies and TKIs. TKIs are small molecules that compete with and prevent binding of adenosine triphosphate to the intracellular tyrosine kinase region. These agents cause tumor regression by increasing apoptosis and inhibiting cellular proliferation and angiogenesis.

### First line and maintenance treatment

Two TKIs (gefitinib and erlotinib) and one monoclonal antibody (cetuximab) have been tested in the first line setting.

Gefitinib (ZD1839, Iressa®), is an oral EGFR-specific anilinoquinazoline which reversibly inhibits autophosphorylation and a shift of cells from S phase to G0/G1 [9]. Two phase III trials in chemotherapy naïve stage IIIB/IV NSCLC patients have so far been reported (INTACT 1 and 2). These multinational, randomized, double blind trials of chemotherapy (gemcitabine plus cisplatin in INTACT 1; paclitaxel plus carboplatin in INTACT 2) in combination with gefitinib or placebo, failed to demonstrate any improvement in OS. In INTACT 1 [10], 1093 patients were included and no difference in efficacy was observed between the treatment arms. For treatment with gefitinib 500 mg/d, 250 mg/d and placebo, median survival was 9.9, 9.9 and 10.9 months, respectively. In INTACT 2 [11], 1037 patients were included in the same three arms with no difference in OS (median 8.7, 9.8, and 9.9 months for gefitinib 500 mg/d, 250 mg/d and placebo, respectively).

Erlotinib (CP-358774, OSI 774, Tarceva®) is another anilinoquinazoline derivative, orally active EGFR inhibitor that can induce both cell-cycle arrest in G1 and apoptosis. Phase I studies led to the determination of a 150 mg per day dose due to its safety profile and pharmacokinetic parameters [12]. In first line, two trials analyzed the combination of erlotinib plus chemotherapy (paclitaxel and carboplatin in the TRIBUTE trial and gemcitabine and cisplatin in the TALENT trial). Both trials failed to show a survival benefit in patients treated with the combination [13,14]. In the TRIBUTE trial, 1059 patients were included with a median survival of 10.6 months and 10.5 months for the erlotinib and placebo arms, respectively. In the TALENT trial, 1172 patients were included with a median survival of 10.75 and 11 months for erlotinib and placebo, respectively.

Erlotinib has been also explored in the maintenance setting after first-line treatment in three randomized trials (SATURN, ATLAS and IFTC-GFPC 0502). The SATURN study demonstrated superior OS of 12.3 months in the erlotinib group vs 11.1 months in the placebo group (HR 0.81; 95% CI: 0.70-0.95; p = 0.0088). Only 21% of patients in the placebo group ever received erlotinib. OS for the EGFR-mutant patients had not been reached for this subgroup receiving erlotinib at time of publication (HR 0.83; 95% CI: 0.34-2.02; p = 0.6810). Patients who had stable disease after first-line chemotherapy seemed to have a more pronounced OS benefit (median 11.9 vs 9.6 months with placebo; HR 0.72; 95% CI: 0.59-0.89; p = 0.0019) than those who responded (median 12.5 vs 12.0 months with placebo; HR 0.94; 95% CI: 0.74-1.20; p = 0.618) [15] Biomarker analysis for *EGFR* mutation status showed that erlotinib was active in patients with *EGFR*-activating mutations (HR 0.10; 95% CI: 0.04-0.25; p < 0.0001) and in those with wild-type *EGFR* (HR 0.78; 95% CI: 0.63-0.96; p = 0.0185). In the ATLAS trial, 743 patients were randomized (1:1) to bevacizumab plus either placebo or erlotinib after four cycles of chemotherapy plus bevacizumab without disease progression or significant toxicity. The primary end point was progression free survival (PFS). Median PFS was 3.7 months with bevacizumab/placebo and 4.8 months with bevacizumab/erlotinib (HR 0.71; 95% CI, 0.58 to 0.86; p < 0.001). There were no differences in OS [16]. IFTC-GFPC0502 investigated whether continuation maintenance with gemcitabine or a switch with erlotinib improves clinical outcome compared with observation in patients with advanced NSCLC whose disease was controlled after cisplatin-gemcitabine induction chemotherapy. PFS was significantly prolonged by gemcitabine (median, 3.8 vs 1.9 months; HR 0.56; 95% CI, 0.44 to 0.72; p < 0.001) and erlotinib (median, 2.9 vs 1.9 months; HR, 0.69; 95% CI, 0.54 to 0.88; p = 0.003) vs observation but neither maintenance strategy resulted in an improvement in OS [17].

Cetuximab (Erbitux®) has also been tested in first line. In a phase III, multinational, multicenter, open-label trial, 1125 advanced NSCLC patients were randomly assigned (1:1) to chemotherapy (cisplatin plus vinorelbine for up to six cycles) plus cetuximab or chemotherapy alone. The primary endpoint was OS. Cetuximab was continued until disease progression or unacceptable toxicity. OS was longer with cetuximab than chemotherapy alone (median 11.3 months vs 10.1 months; HR 0.871; 95% CI 0.762-0.996; p = 0.044) with no differences in PFS [18].

### Second line and beyond

EGFR TKIs have also been tested following first line treatment for advanced NSCLC. Two large phase II, randomized trials (IDEAL 1–2) have been reported using gefitinib. In the IDEAL 1 study [19], 210 NSCLC patients who had failed one or two chemotherapy regimens (at least one platinum-based therapy) were randomly assigned to receive 250 mg or 500 mg per day of gefitinib. In the IDEAL 2 study [20], 216 patients who had failed two or more chemotherapy regimens containing platinum and docetaxel received 250 mg or 500 mg gefitinib per day. There were no differences between the two doses with regard to response rate, time to progression or median survival. An increased incidence of adverse events in patients receiving 500 mg per day was seen in both trials. Three phase III trials have been performed in this setting with gefitinib. ISEL is a randomized, placebo controlled, phase III trial of gefitinib in chemotherapy-refractory NSCLC patients. Median survival was 5.6 months in the gefitinib group and 5.1 months in the placebo group, failing to demonstrate a benefit [21]. The V-15-32 study enrolled 484 Japanese patients in a non-inferiority trial that compared OS with gefitinib vs docetaxel in NSCLC patients who had failed one or two chemotherapy regimens. Non-inferiority in OS was not achieved (HR 1.12; 95% CI, 0.89 to 1.40) [22]. In the INTEREST phase III trial, 1466 patients were enrolled in second line. The results of this study demonstrated non-inferiority of gefitinib vs docetaxel in terms of OS with a median 7.6 and 8.0 months (HR 1.020, 95% CI 0.905-1.150). Gefitinib was better tolerated and quality of life evaluation favored its use [23].

Erlotinib has been evaluated following first line treatment in three trials. The BR.21 trial enrolled advanced NSCLC patients with performance status 0 to 3 and randomized them to receive erlotinib or placebo in second or third line. The trial was the first to demonstrate activity of an EGFR TKI in NSCLC with response rate of 8.9% in the erlotinib group and less than 1% in the placebo group (p < 0.001). PFS was 2.2 months and 1.8 months, respectively (HR 0.61; p < 0.001). OS was 6.7 months with erlotinib and 4.7 months with placebo (HR 0.70; p < 0.001) [24]. On the basis of these results, erlotinib was approved by the FDA in November 2004 and by the EMEA in October 2005 for second and third line treatment of NSCLC patients. TAILOR is a randomized controlled trial in 52 Italian hospitals in advanced EGFR wild-type NSCLC patients who had received platinum-based chemotherapy. Seven hundred and two patients were screened and 222 were enrolled to receive either erlotinib or docetaxel. Median OS was 8.2 months (95% CI 5.8-10.9) with docetaxel vs 5.4 months (95% CI 4.5-6.8) with erlotinib (HR 0.73, 95% CI 0.53-1.00; p=0.05). Median PFS was 2.9 months (95% CI 2.4-3.8) with docetaxel vs 2.4 months (95% CI 2.1-2.6) with erlotinib (HR 0.71, 95% CI 0.53-0.95; p = 0.02) [25]. The TITAN trial was a randomized, open-label, phase III study carried out at 77 sites in 24 countries. Four hundred and twenty four NSCLC patients with disease progression after chemotherapy were randomly assigned to receive erlotinib or chemotherapy (standard docetaxel or pemetrexed). Median OS was 5.3 months (95% CI 4.0-6.0) with erlotinib and 5.5 months (95% CI 4.4-7.1) with chemotherapy (HR 0.96, 95% CI 0.78-1.19; p = 0.73) [26].

### Targeting EGFR in selected populations: EGFR mutations

Since only 8.9% of unselected NSCLC patients responded to EGFR TKI [24], several studies have tried to find prognostic and predictive biomarkers of sensitivity or resistance to anti-EGFR agents. For example: EGFR mutations, *EGFR* gene copy numbers, status of EGFR ligands, changes in other *HER* family genes or molecules downstream to EGFR including KRAS or AKT. However, only EGFR mutations have been demonstrated a strong correlation with efficacy of EGFR TKIs in different prospective trials.

In 2004, three different groups found that a subset of NSCLC patients have somatic, activating mutations of the *EGFR* gene [27-29]. Following these initial reports, various groups have confirmed and extended the findings. Mutations in this gene in lung cancer have been located in four exons, from 18 to 21, which encode the kinase domain [30]. More than 188 EGFR mutations have been reported but just two - deletion of 5 amino acids from exon 19 and the missense mutation in exon 21 resulting in a substitution of arginine for leucine at position 858 (L858R) - account for 80-90% [31]. These activating mutations occur in the ATP binding domain, leading to a constitutively active receptor. Importantly, these changes also lead to superior binding of the domain to targeted TKI compared with ATP [32,33]. Several less common mutations, such as G719X, L861X, and insertions at exon 19, have demonstrated drug sensitivity, whereas others appear less responsive (such as, EGFR exon 20 insertion mutations). These molecular alterations are most common in Asian patients, adenocarcinoma histology, female gender and never smokers, who are known to be candidates for harbor EGFR mutations [34,35].

The IPASS was the first phase III trial to analyze the efficacy of an EGFR TKI in EGFR-mutant NSCLC patients, selected according to clinical characteristics. This open-label, phase III study randomized 1217 previously untreated, advanced NSCLC, non smokers or former light smoker patients from East Asia with adenocarcinoma histology to receive gefitinib or carboplatin plus paclitaxel. The study met its primary objective of non inferiority of gefitinib. A total of 683 patients (56.1%) provided samples. *EGFR* mutation data for 437 patients (35.9%) was evaluated. Of the 437 samples, 59.7% were positive for a mutation: 140 (53.6%) had exon 19 deletions; 111 (42.5%) had a mutation at exon 21 (L858R). In the subgroup of 261 mutation positive patients, PFS was significantly longer among those who received gefitinib than chemotherapy (HR 0.48; 95% CI, 0.36 to 0.64; p < 0.001), whereas in the subgroup of 176 patients negative for the mutation, PFS was significantly longer among those who received carboplatin-paclitaxel (HR 2.85; 95% CI, 2.05 to 3.98; p < 0.001). A higher objective response rate (RR) (71.2% vs 47.3% with chemotherapy) was observed among patients who received gefitinib [36].

In addition to the IPASS findings, there have been six randomized controlled phase III trials comparing EGFR TKIs (gefitinib, erlotinib or afatinib) to chemotherapy in *EGFR*-mutant lung cancer patients, both in Asian and Caucasian populations (Table 1). These studies uniformly show superior RRs, PFS and quality of life with EGFR TKIs compared to chemotherapy. No differences in OS were shown, except for with afatinib. In the WJTOG3405 phase III trial, 177 chemotherapy-naïve, EGFR mutated (either exon 19 deletion or L858R point mutation), advanced NSCLC patients from 36 centers in Japan were randomly assigned to receive either gefitinib or cisplatin plus docetaxel for three to six cycles. The gefitinib arm had significantly longer PFS compared with the cisplatin plus docetaxel group, 9.2 months (95% CI 8.0-13.9) vs 6.3 months (9% CI 5.8-7.8; HR 0.489, 95% CI 0.336-0.710, p < 0.0001) [37]. The NEJ002 study included 230 patients enrolled from 43 centers in Japan. PFS was significantly longer in the gefitinib group, 10.8 months, vs 5.4 months with chemotherapy (HR, 0.30; 95% CI, 0.22 to 0.41; p < 0.001). The objective RR was significantly higher in the gefitinib group (73.7% vs. 30.7%, p <0.001). OS did not differ significantly between the two treatment groups [38].

**Table 1:** **Phase III trials of EGFR TKIs versus chemotherapy as first line therapy in NSCLC EGFR mutated patients**

**Study**	**TKI**	**Chemotherapy**	**Population**	**Response rate (EGFR TKI vs chemotherapy) %**		**Disease control rate (EGFR TKI vs chemo) %**		**Progression free survival (EGFR TKI vs chemo) m**		**Overall survival (EGFR TKI vs chemo) m**	
**IPASS (Sub-study)** ^**1**^	Gefitinib	Carbo/Pac	Asian (E/SE)	71.2	47.3	91.7	87.6	9	6	21.6	21.9
				p 0.001				p 0.001		NS	
**EURTAC** ^**2**^	Erlotinib	Cis OR Carbo + Doc OR Gem	Caucasian	58	15	79	66	9.4	5.2	NR	NR
				p < 0.001		p <0.001		p < 0.0001			
**OPTIMAL** ^**3**^	Erlotinib	Carbo/Gem	Asian (China)	83	36	96	82	13.1	4.6	NR	NR
				p 0.00001		p <0.0001		p < 0.0001			
**WJTOG 3405** ^**4**^	Gefitinib	Cis/Doc	Asian (Japan)	62.1	32.2	93.1	78	9.2	6.3	30.9	NR
				p < 0.0001		p 0.02		p < 0.0001		p 0.211	
**NEJSG 002** ^**5**^	Gefitinib	Carbo/Pac	Asian (Japan)	73.7	30	86	79	10.8	5.4	30.5	23.6
				p < 0.001				p < 0.001		p 0.353	
**LUX-Lung 3** ^**6**^	Afatinib	Cis/Pem	Majority Asian	56.1	22.6	NR	NR	11.1	6.9	31.6	28.2
				p < 0.0001				p 0.0004		p 0.109	
**LUX-Lung 6** ^**7**^	Afatinib	Cis/Gem	Asian (E/SE)	67	23	93	76	11	5.6	23.6	23.5
				p < 0.0001		p < 0.0001		p 0.001		p0.175	

^1^Mok, et al. N Engl J Med 2009 [36]; ^2^; ^2^Rosell, et al. Lancet Oncol 2012 [40]; ^3^Zhou, et al. Lancet Oncol 2011 [39]; ^4^Mitsudomi, et al. Lancet Oncol 2010 [37]; ^5^Maemondo, et al. N Engl J Med 2010 [38]; ^6^Sequist, et al. J Clin Oncol 2013 [43]; ^7^Wu, et al. ASCO 2013 [44].

Two trials, the OPTIMAL and the EURTAC, have been carried out in this setting with erlotinib. The OPTIMAL study was an open-label, randomized, phase III trial involving 22 centers in China. One hundred and sixty five patients were randomly assigned to receive either erlotinib or carboplatin plus gemcitabine. Median PFS was 13.1 months (95% CI 10.58-16.53) in erlotinib-treated patients vs 4.6 months (4.21-5.42) (HR 0.16, 95% CI 0.10-0.26; p < 0.0001). Overall response rate (ORR) was 83% (68/82) for erlotinib and 36% for chemotherapy (P < 0.0001) [39]. The EURTAC trial was an open-label, randomized phase III study conducted in 42 hospitals in Spain, France and Italy and is the only trial to date in a Caucasian population. EGFR-mutant NSCLC patients were randomized to erlotinib or standard chemotherapy with cisplatin or carboplatin plus docetaxel or gemcitabine. The primary endpoint was PFS. One thousand two hundred and twenty seven patients were screened and 174 with EGFR mutations enrolled. The preplanned interim analysis showed that the study met its primary endpoint: median PFS was 9.7 months (95% CI 8.4-12.3) in the erlotinib group, compared with 5.2 months (95% CI 4.5-5.8) in the chemotherapy group (HR 0.37, 95% CI 0.25-0.54; p < 0 0001) [40].

Afatinib (BIBW 2992, Gilotrif®, Giotrif®,) is an irreversible, pan-HER inhibitor that blocks all members of the HER family with tyrosine kinase properties (EGFR, HER2, and HER4). It is an ATP-competitive aniline-quinazoline compound with a reactive acrylamide group that irreversibly binds to cysteine residues within the kinase domain of EGFR and HER2 [41]. Moreover, afatinib is able to inhibit kinase activity in vitro and in animal models when a resistant T790M mutation is concomitant to a sensitive mutation. The LUX-Lung 2 was a single arm phase II trial that assessed activity of two doses of afatinib (40 mg and 50 mg daily) as first- or second-line treatment in 129 NSCLC patients harboring *EGFR* mutations. Ninety nine patients started afatinib at 50 mg and 30 patients at 40 mg. ORR was 61%, with no significant differences according to the dose. As no apparent difference in activity was highlighted between the two groups of patients, 40 mg dosing was chosen for phase III trials. Median PFS was 10.1 months and OS 24.8 months for all patients [42]. LUX-Lung 3 and LUX-Lung 6 were phase III trials in first line with afatinib vs chemotherapy in this population. LUX-Lung 3 randomized (2:1) 345 patients to receive afatinib or a combination of cisplatin and pemetrexed. This trial included 27% non-Asians patients and patients with uncommon mutations. Median PFS was 11.1 months for afatinib compared to 6.9 months for chemotherapy (HR: 0.58; 95% CI: 0.43–0.78; p = 0.001). Better PFS of 13.6 months was shown in patients with common activating mutations (exon 19 deletions and exon 21 point mutations) [43]. The LUX-Lung 3 is a phase III trial with a similar design to LUX-Lung 6 in 364 Asian patients. Patients in the control arm received cisplatin plus gemcitabine. Patients receiving afatinib compared to chemotherapy had significantly prolonged PFS (11 vs 5.6 months, HR 0.28, 95% CI: 0.20–0.39; p = 0.0001) as well as higher ORR ( 66.9 vs 23%; *p =* 0.0001) [44]. A clinical trial comparing afatinib to gefitinib is already ongoing (LUX-Lung 7) but no data are yet available. However, EGFR TKIs had not demonstrated a benefit in OS until ASCO 2014. OS results for LUX-Lung 6 and 3 were presented at the ASCO annual congress 2014. The studies showed median OS for common mutations of 27.3 months in the afatinib arm vs 24.3 months in the chemotherapy arm (HR 0.81; 95% CI 0.66-0.99; p = 0.0374) [45].

**Figure 2:**
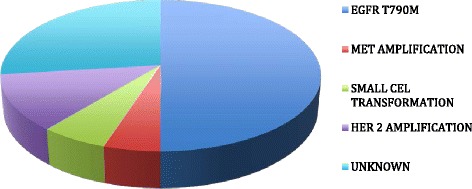
**Mechanisms of acquired resistance to EGFR-tyrosine kinase inhibitor.**

Despite impressive results in RR and PFS, clinical failure is eventually inevitable in these patients. Serial biopsies of tumors taken before and after EGFR TKI treatment have provided insight into the mechanisms of treatment failure [46] (Figure 2). In around 60% of cases, this is mediated by the presence of the secondary *EGFR* T790M mutation resistant to inhibition by current EGFR TKIs; however, other mechanisms have also been described [47,48]. Different strategies could be applied beyond progression, such as addition of chemotherapy, switching to chemotherapy or continuing EGFR TKI.

Recently, third-generation EGFR inhibitors, such as WZ4002, CO-1686, HM61713 and AZD9291, have been developed and interesting results from phase I trials were presented at ASCO 2014 [49,50].

## Conclusions

At last, a revolution is taking place in lung cancer treatment. The discovery of driver mutations and their treatment has changed our understanding of cancer. The EGFR pathway plays an important role in lung cancer, especially EGFR mutations which have opened up the possibility of a personalized medicine approach to this disease. EGFR TKIs are a treatment option in unselected populations after first line, though this remains controversial. However, EGFR TKIs are now standard treatment in EGFR-mutant patients, where phase III trials have demonstrated a clear efficacy benefit. There still remains much work to do in this setting, and, with ever-growing knowledge, we are likely to see fundamental changes in clinical practice in the coming years.
